# Characterization of TRPA channels in the starfish *Patiria pectinifera*: involvement of thermally activated TRPA1 in thermotaxis in marine planktonic larvae

**DOI:** 10.1038/s41598-017-02171-8

**Published:** 2017-05-19

**Authors:** Shigeru Saito, Gen Hamanaka, Narudo Kawai, Ryohei Furukawa, Jun Gojobori, Makoto Tominaga, Hiroyuki Kaneko, Yoko Satta

**Affiliations:** 1 Division of Cell Signaling, Okazaki Institute for Integrative Bioscience (National Institute for Physiological Sciences), National Institute of Natural Sciences, Okazaki, Aichi 444-8787 Japan; 2 Department of Physiological Sciences, SOKENDAI (The Graduate University for Advanced Studies), Okazaki, Aichi 444-8787 Japan; 3 Tateyama Marine Laboratory, Marine and Coastal Research Center, Ochanomizu University, Kouyatsu, Tateyama, Chiba, 294-0301 Japan; 4 Department of Biology, Research and Education Center for Natural Sciences, Keio University, Hiyoshi, Kouhoku-ku, Yokohama, Kanagawa 223-8521 Japan; 5 Division of Biomedical Information Analysis, Iwate Tohoku Medical Megabank Organization, Iwate Medical University Disaster Reconstruction Center, Nishitokuda, Yahaba-cho, Shiwa-gun, Iwate, 028-3694 Japan; 6 Department of Evolutionary Studies of Biosystems, School of Advanced Sciences, SOKENDAI (The Graduate University for Advanced Studies), Hayama, Kanagawa 240-0193 Japan

## Abstract

The vast majority of marine invertebrates spend their larval period as pelagic plankton and are exposed to various environmental cues. Here we investigated the thermotaxis behaviors of the bipinnaria larvae of the starfish, *Patiria pectinifera*, in association with TRPA ion channels that serve as thermal receptors in various animal species. Using a newly developed thermotaxis assay system, we observed that *P. pectinifera* larvae displayed positive thermotaxis toward high temperatures, including toward temperatures high enough to cause death. In parallel, we identified two *TRPA* genes, termed *PpTRPA1* and *PpTRPA basal*, from this species. We examined the phylogenetic position, spatial expression, and channel properties of each *Pp*TRPA. Our results revealed the following: (1) The two genes diverged early in animal evolution; (2) *PpTRPA1* and *PpTRPA basal* are expressed in the ciliary band and posterior digestive tract of the larval body, respectively; and (3) *Pp*TRPA1 is activated by heat stimulation as well as by known TRPA1 agonists. Moreover, knockdown and rescue experiments demonstrated that *Pp*TRPA1 is involved in positive thermotaxis in *P. pectinifera* larvae. This is the first report to reveal that TRPA1 channels regulate the behavioral response of a marine invertebrate to temperature changes during its planktonic larval period.

## Introduction

Unlike the adult phase, during which many animals concentrate on their reproductive activity, in the larval phase, animals concentrate on optimizing growth and survival. The majority of marine animals have a free-floating zooplanktonic larval phase (34 of the approximately 40 animal phyla)^1^. Marine planktonic larvae are exposed to dynamic changes in a wide variety of abiotic conditions such as acidification^2^, salinity^3^, flow^4^, viscosity^5^, light^6^, and temperature^7, 8^, and larvae may develop certain physiological functions in response to these environmental cues.

The starfish is an echinoderm that is phylogenetically close to the chordates. There are approximately three hundred genera of starfish, which include approximately 1,900 extant species^9^. One species of starfish, *Patiria pectinifera*, has a remarkably wide distribution in the marine areas of Japan^10–12^. Such phylogenetic and ecological prosperities of the starfish should (or might) depend on the physiological functions of the larvae, which are acquired in the course of evolution and are valuable for the growth of *P. pectinifera* into adults. However, how starfish larvae behave in response to environmental factors is poorly understood. In our preliminary experiments (unpublished data), larvae of the starfish *P. pectinifera* appeared to be attracted to light. By using infrared or visible light isolation filters, we further found that starfish larvae are attracted to infrared radiation, suggesting that they possess an ability to perform positive thermotaxis toward warmth. These findings provide the opportunity to investigate how planktonic larvae actively respond to environmental cues such as temperatures.

Thermosensory systems play a crucial role in thermotaxis. Molecular identification of thermal sensors has enabled us to examine the molecular mechanisms driving thermotaxis behaviors^13^. Many studies have focused on transient receptor potential (TRP) ion channels as the key molecules in thermosensory systems essential for the survival of various organisms^14–16^. These molecules are called thermoTRP channels, and they play pivotal roles in initiating signal transduction of thermal perception. TRP channels are non-selective cation channels that have six transmembrane domains with intracellular N- and C-terminal regions^17, 18^. In mammals, ten thermoTRP channels have been identified and further classified into three subfamilies (TRPV, TRPM, and TRPA)^14–16^, and several homologous genes to these thermoTRP channels have been identified in a wide variety of animal species including vertebrates, insects, and even cnidarians^19–22^.

In the three TRP subfamilies, *TRPA* genes from various animals have been investigated with respect to their molecular structure, function, and phylogenetic relationships. Phylogenetic analyses of available *TRPA* sequences have shown that *TRPA* genes can be grouped into two clades: TRPA1 and basal TRPA. Genes in the TRPA1 clade are shared between vertebrates and invertebrates, while homologs in the basal TRPA clade are only found in invertebrates^19^. In addition, thermoTRPA channels have attracted considerable interest given that they serve as thermal receptors in both vertebrates and *Drosophila* ^19, 23–31^. For example, TRPA1 is potentially activated by cold temperatures in rodents (<17 °C)^29^, while it is activated by warm temperatures in *Drosophila* (>24–29 °C)^31^. These channels are also activated by several irritant chemical compounds, thus serving as multimodal receptors^14–16^. The relationship between thermal TRPA1 channels and thermotaxis has mainly been investigated in insects such as fruit flies, e.g., *Drosophila melanogaster*, and TRPA1 has been reported to be involved in heat avoidance in other species^25, 32^. Despite an accumulation of a limited number of such model organisms, the functional properties of TRP channels have yet to be examined in non-model organisms, especially in marine planktonic larvae.

In the present study, we aimed to examine thermotaxis in 4-day-old bipinnaria larvae of the starfish *P. pectinifera*, in association with TRPA ion channels. Here, we developed a novel accumulation assay system with thermal gradients to examine larval thermotaxis. In parallel, we report the first identification of two *TRPA* genes, termed *PpTRPA1* and *PpTRPA basal*, in *P. pectinifera*. We performed a series of analyses to characterize *PpTRPA1* and *PpTRPA basal* in terms of phylogeny, spatial expression, and channel sensitivity to temperature and chemical stimuli. We found that *PpTRPA1* and *PpTRPA basal* are expressed differently in *P. pectinifera* larvae: the former is expressed in the ciliary band, and the latter is concentrated in the posterior region of the digestive tract. Interestingly, *Pp*TRPA1 is activated by heat stimulation as well as known TRPA agonists. Based on these results, we conducted both a loss-of-function experiment on *PpTRPA1* with morpholino antisense oligonucleotides and its rescue experiment with synthetic *PpTRPA1* complementary RNA. Our results suggest that *Pp*TRPA1 is involved in positive thermotaxis in *P. pectinifera* larvae.

## Results

### Movement of starfish larvae in thermal gradients

**Figure 1:**
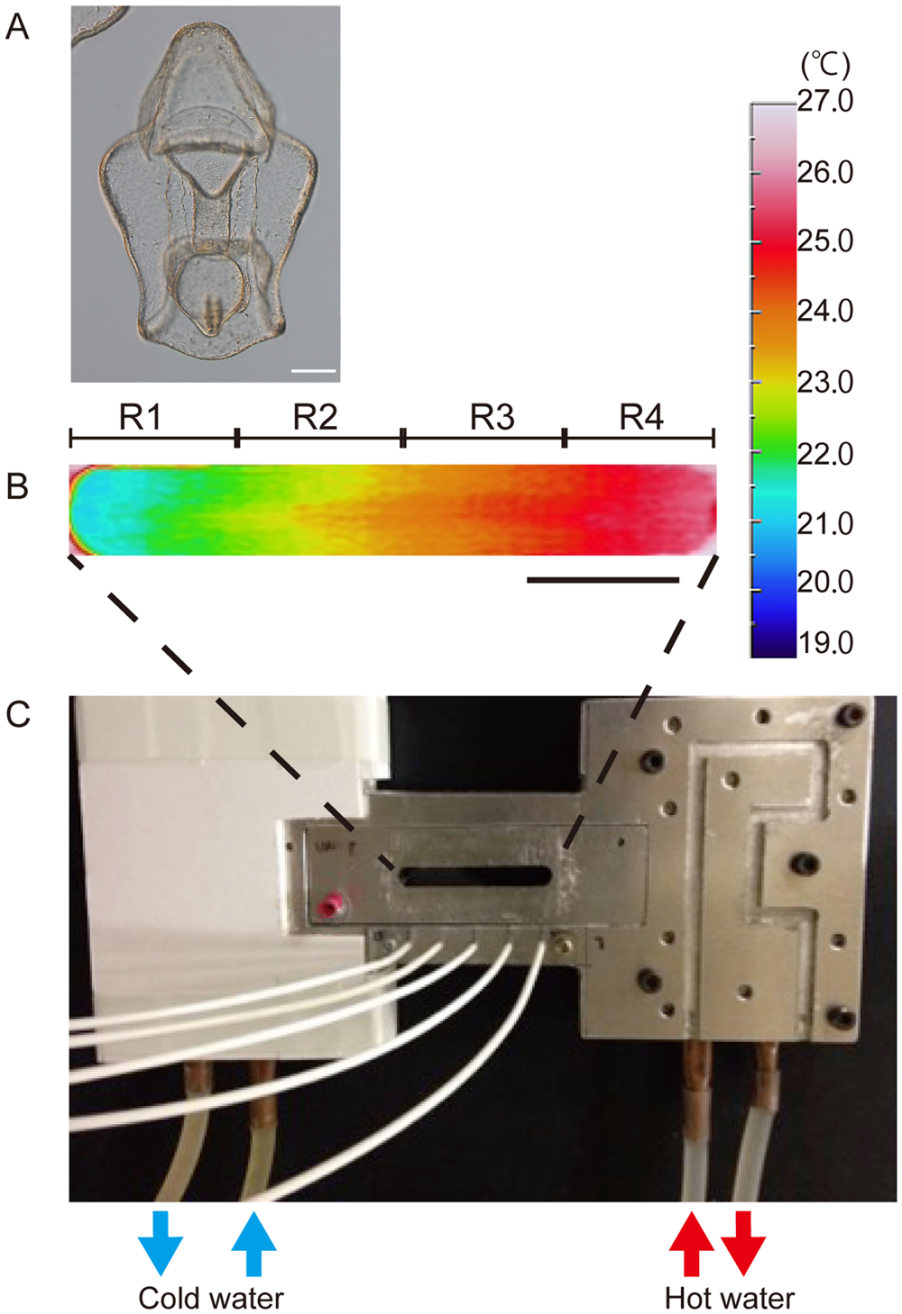
Thermotaxis assay system for starfish larva. (**A**) A 4-day-old-bipinnaria larvae of the starfish, *P. pectinifera*. Scale; 100 µm. (**B**) Thermal image of a representative 20–25 °C thermal gradient. The image was taken 15 min after placing the test chamber on the thermal stage. No obvious changes in the gradient were observed during the experiment. Scale; 1 cm. (**C**) Overview of the thermotaxis assay system. Five thermal sensors (white cables) were placed beneath the chamber and used to monitor temperature throughout the assay. Temperatures measured with the thermal sensors (shown in Fig. 2A) were essentially the same as those obtained by thermography as shown in Fig. 1B.

Due to our preliminary experiments suggesting that *P. pectinifera* larvae possess the ability to perform positive thermotaxis, we developed an accumulation assay apparatus suitable for examining larval thermotactic movement (Fig. 1). The chamber was filled with seawater, and both ends were cooled or heated to form the desired thermal gradient. The temperature of the chamber was monitored by five thermal sensors that were evenly placed beneath the chamber as shown in Fig. 1C. Furthermore, thermographic images of the chamber were taken for each of the thermal gradients: 15–20 °C, 20–25 °C, and 25–30 °C. Figure 1B shows a typical image for the 20–25 °C thermal gradient. The temperature distributions obtained from the thermographic images and those obtained by the thermal sensors were consistent. In each assay, the thermal gradient was formed within 3 min after setting up the chamber (Fig. 1C, see Materials and Methods) and persisted without mixing for at least 60 min even when 100 larvae were released into the chamber. Thermal convection was negligible when the chamber temperatures were thermographically observed. Using this experimental setup, we analyzed thermotactic movement of approximately 50 bipinnaria larvae (4-day-old larvae). We divided the chamber into four equal areas, which were delineated by five thermosensors (Fig. 1B; R1, R2, R3, R4), and counted the number of larvae in each area at 0, 30, and 60 minutes after releasing larvae.

**Figure 2:**
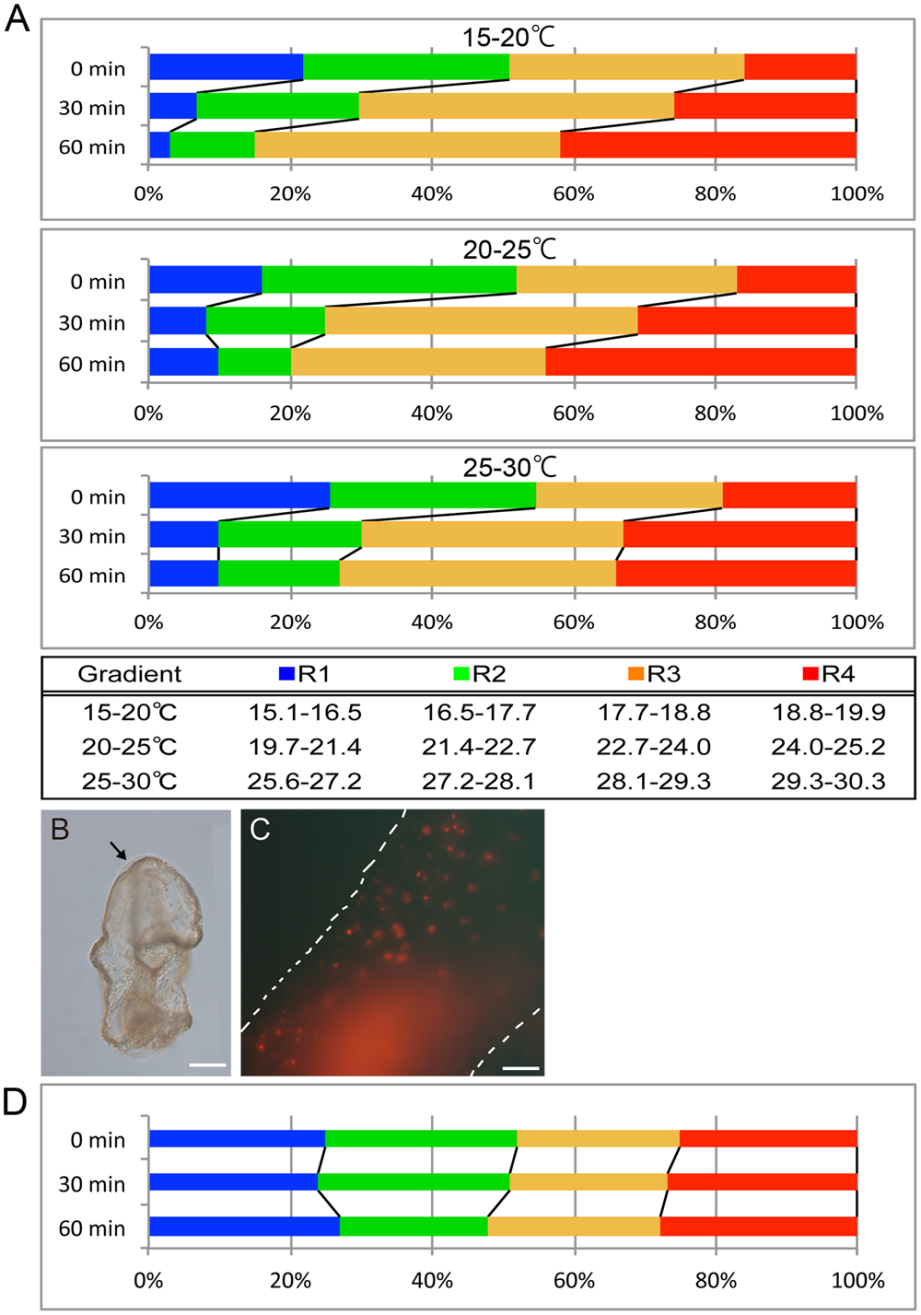
Thermotaxis assay of starfish larvae and the inhibitory effect on thermotaxis by a broad TRP channel blocker. (**A**) Changes in the number of larvae in each temperature range over time (n = 3). In each assay, approximately 50 larvae were added to the chamber. Larvae showed positive thermotaxis toward higher temperatures in each thermal gradient treatment. The table shows the upper and lower temperatures for each area (the chamber was equally divided into 4 areas) indicated by different colors according to the thermal gradient images in Fig. 1B. Proportions of larvae in each area are shown at 0, 30, and 60 min after the onset of the assay. (**B**) Morphology of a bipinnaria larva that stopped swimming at approximately 33 °C in the 31–38 °C thermal gradient. This photograph was taken at 10 min after the initiation of the assay. The arrow shows the area where the extracellular matrix (ECM) had separated from the epithelial cells. Scale; 100 µm. (**C**) High magnification image of the ectodermal epithelial cells in (**B**). Propidium iodide (PI) permeates into the nuclei of larval cells. White dotted lines represent the outline of the larval body. Scale; 20 µm. (**D**) Thermotaxis assay in the 20–25 °C thermal gradient with ruthenium red (RR, 20 µM)–treated larvae (n = 3). RR–treated larvae did not exhibit thermotactic behaviors as shown in A (middle).

Supplemental Movie 1 shows a typical thermotaxis assay in the 20–25 °C thermal gradient. In the 15–20 °C and 20–25 °C gradients, larvae gradually accumulated at higher temperatures over time (Fig. 2A; n = 3, i.e., three independent experiments for each temperature condition), suggesting that larvae perform positive thermotaxis. In contrast, when the chamber temperature was maintained constant at 20 °C, larvae did not exhibit any positive thermotaxis (Supplementary Fig. S1, Supplementary Movie 2). In the 25–30 °C gradient, the number of larvae in the highest temperature area reached a plateau at 30 min after the onset of experiments (n = 3). Notably, in the 26–36 °C thermal gradient, the larvae swam up the gradient but paused at around 33 °C (Supplemental Movie 3). In this case, these larvae lost transparency and became opaque. In addition, part of the outer layer of the extracellular matrix (ECM) of the larvae detached from the epithelial body wall (Fig. 2B, see also Fig. 1A) thereby becoming permeable to propidium iodide (PI) which is usually used to stain dead cells (Fig. 2C). Thus, *P. pectinifera* larvae exhibited positive thermotaxis toward high, deleterious temperatures and eventually most larvae stopped swimming and died in the 26–36 °C thermal gradient when they reached temperatures greater than 33 °C.

To examine whether thermoTRP channels were involved in the thermotactic movements of larvae, we conducted accumulation assays in the presence of ruthenium red (RR), a general antagonist for various TRP channels^16^. As shown in Fig. 2D and Supplemental Movie 4, no temporal change in accumulation ratio was observed throughout the 60 min period in the 20–25 °C gradient, although larvae kept swimming during the assay. Similar results were obtained in the 15–20 °C and 25–30 °C thermal gradients (data not shown). These results indicate the possibility that some TRP channels were expressed in *P. pectinifera* larvae and functioned as thermosensors for positive thermotaxis toward warmth.

### Molecular structure of TRPA genes and their phylogenetic features

**Figure 3:**
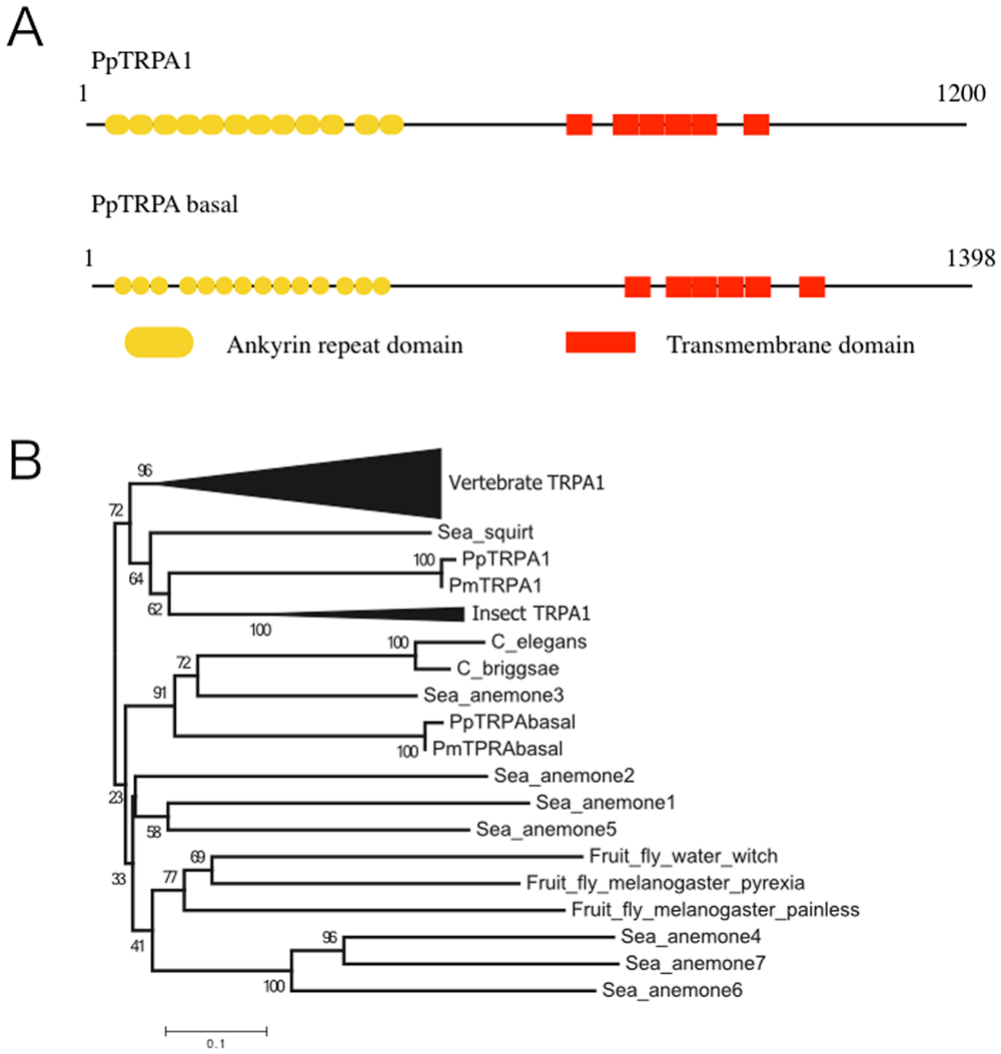
Domain structures and phylogenetic positions of *PpTRPA1* and *PpTRPA basal*. (**A**) Schematic drawing of *PpTRPA1* and *PpTRPA basal*. The length of the amino acid sequences is indicated just above the lines. (**B**) The NJ-tree including known TRPA channels and *PpTRPA1* and *PpTRPA basal*. Only the amino acid sequences of the transmembrane regions were used for phylogenetic analysis. Bootstrap values obtained from 1,000 replications are shown near the nodes. Because complete deletion reduces the number of amino acids, the pair-wise deletion option for calculation of genetic distances was used. The average number of amino acids used was 225.2.

To understand the molecular mechanisms driving thermotaxis in *P. pectinifera* larvae, we focused on the TRPA channel subfamily as several of these channels have been shown to serve as thermal receptors in other invertebrate species^23, 25, 28, 30, 31^. Because many invertebrates have multiple copies of *TRPA* ^19^, we speculated that starfish might have multiple copies of this gene as well^33^. To determine the number of *TRPA* copies in the starfish genome, we searched the available *Patiria miniata* whole genome DNA sequence for *TRPA* genes using HMMER with a profile based on known *TRPA* genes^34^. We found two *TRPA*-like genes in the *P. miniata* genome. In addition, in the transcriptome analysis of *P. pectinifera* (see Materials and Methods), two *TRPA*-like genes were also detected. These *TRPA*-like sequences were grouped into two possible orthologous gene pairs. One copy of the *TRPA*-like gene possessed 1,200 amino acids in its putative coding sequence while the other copy had 1,398 amino acids in its putative coding sequence (Fig. 3A). We searched for conserved domains in the two genes from *P*. *pectinifera* using the Pfam database^35^. Both genes were characterized by several ankyrin repeat domains in the N-terminal region and six-transmembrane domains in the middle. These features were shared with the known TRPA1 and we conclude that these *TRPA*-like genes had domain structures typical of the TRPA subfamily.

We constructed the NJ-tree using known *TRPA* genes with newly found genes from two *Patiria* species (Fig. 3B). The phylogenetic tree obtained coincided with one produced by Kang *et al*.^19^ who suggested that the *TRPA* homologs could be divided into the TRPA1 and basal clades. The TRPA1 clade contained genes from both deuterostomes and protostomes, showing that the two clades had diverged in a common ancestor of deuterostomes and protostomes. Our tree showed that the shorter copies of *TRPA*-like genes from *P. pectinifera* and *P. miniata* were included in the TRPA1 clade; we named these copies *PpTRPA1* and *PmTRPA1*, respectively. The longer copies of the *TRPA*-like genes from *P. pectinifera* and *P. miniata* were included in the basal clade; thus, we named these copies *PpTRPA basal* and *PmTRPA basal*, respectively.

### Expression of *PpTRPA1* and *PpTRPA basal* in *P. pectinifera* larvae

**Figure 4:**
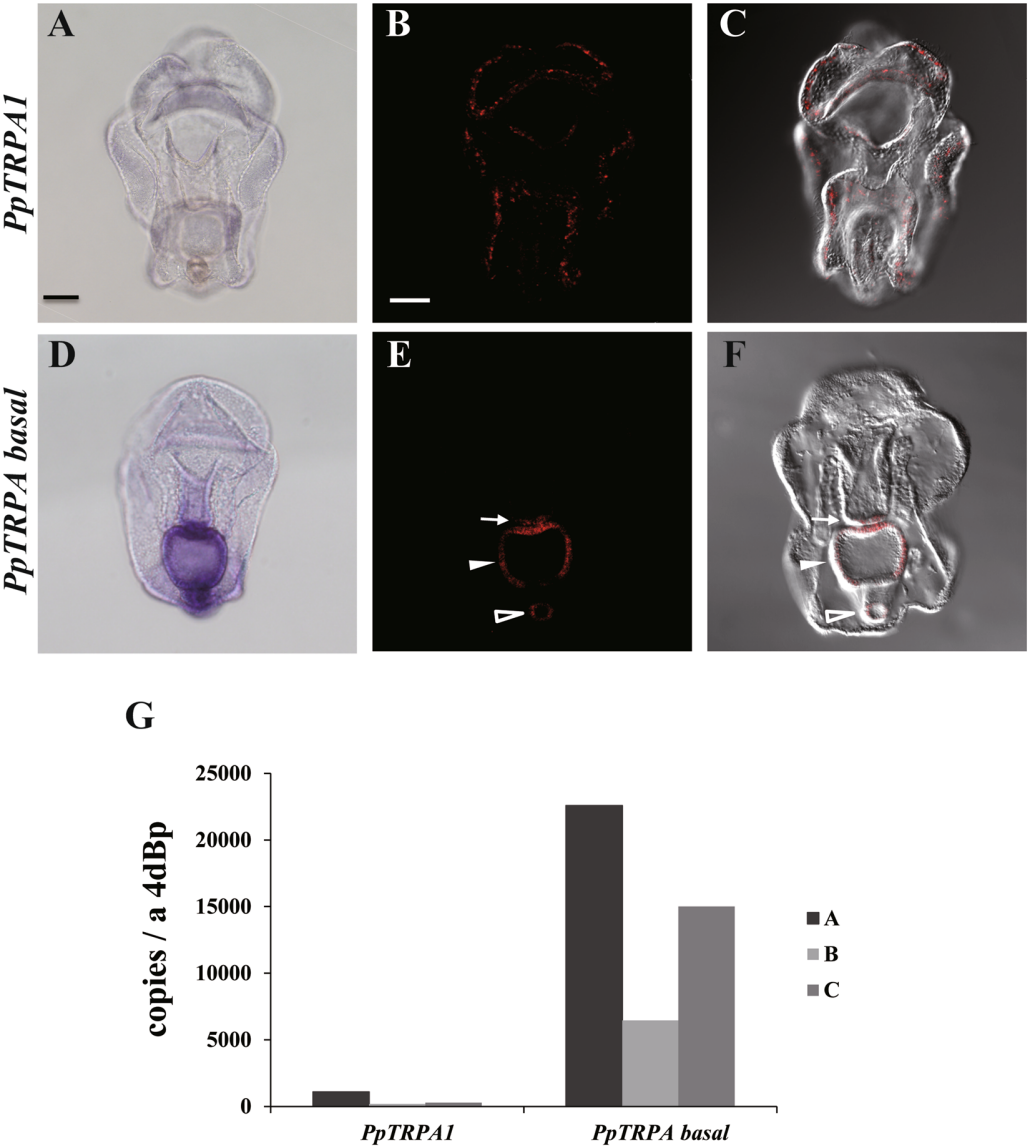
The expression patterns of *PpTRPA1* and *PpTRPA basal* genes at the bipinnaria stage. (**A–F**) Whole mount *in situ* hybridization (WISH) illustrating gene expression patterns of (**A–C**) *PpTRPA1* and (**D–F**) *PpTRPA basal* at the bipinnaria stage as blue or fluorescent red signals, which were detected by NBT-BCIP or HNPP, respectively. Larvae are shown in the ventral view. (**B**, **C**) Images were created with the maximum-intensity method (**B**) using z-stack acquired by the confocal microscopy and (**C**) merged with blight field. (**E**, **F**) Coronal-sectioned images were acquired by the confocal microscopy and then (**F**) merged with blight field. (**E**) Open arrowhead, arrow, and filled arrowhead indicate the intestine, posterior end of the esophagus, and stomach of the bipinnaria larva, respectively. The scale bar is 50 µm. (**G**) Expression values of *PpTRPA1* and *PpTRPA basal* determined by quantitative PCR analysis at the bipinnaria stage. The y-axis shows the number of copies per larva. Three independent batches were used to extract total RNA from the bipinnaria stage. 4dBp: 4-day-old bipinnaria.

The spatial expression patterns for both *PpTRPA1* and *PpTRPA basal* were examined by whole-mount *in situ* hybridization (WISH) in 4-day-old larvae. Using NBT (nitro blue tetrazolium)-BCIP (5-bromo-4-chloro-3-indolyl-phosphate), *PpTRPA1* was observed as a slightly blue-colored signal in the ciliary band (Fig. 4A). This signal became obvious when using fluorescent WISH with HNPP (2-hydroxy-3-naphthoic acid-2′-phenylanilide phosphate) (see Materials and Methods). i.e., the fluorescent signals were widely dispersed in the ciliary band (Fig. 4B,C). Faint signals were also detected in the intestine. In the controls, which were performed with a sense probe of *PpTRPA1*, no signals were detected (Supplementary Fig. S2). On the other hand, by using NBT-BCIP, the expression of *PpTRPA basal* was clearly detected in the posterior region of the digestive tract (Fig. 4D). Under fluorescent WISH with HNPP, the *PpTRPA basal*-signal was clarified, showing it to be densely distributed in the posterior end of the esophagus, stomach, and intestine (Fig. 4E,F).

Next, the expression levels of the *PpTRPA1* and *P*p*TRPA basal* genes were examined by qPCR. As shown in Fig. 4G, transcripts for both of the genes were detected in the bipinnaria larvae with the expression levels considerably lower in *PpTRPA1* than in *PpTRPA basal*. Thus, two genes belonging to the TRPA subfamily in *P. pectinifera* differed in terms of tissue distribution and expression level.

### Channel properties of *Pp*TRPA1

**Figure 5:**
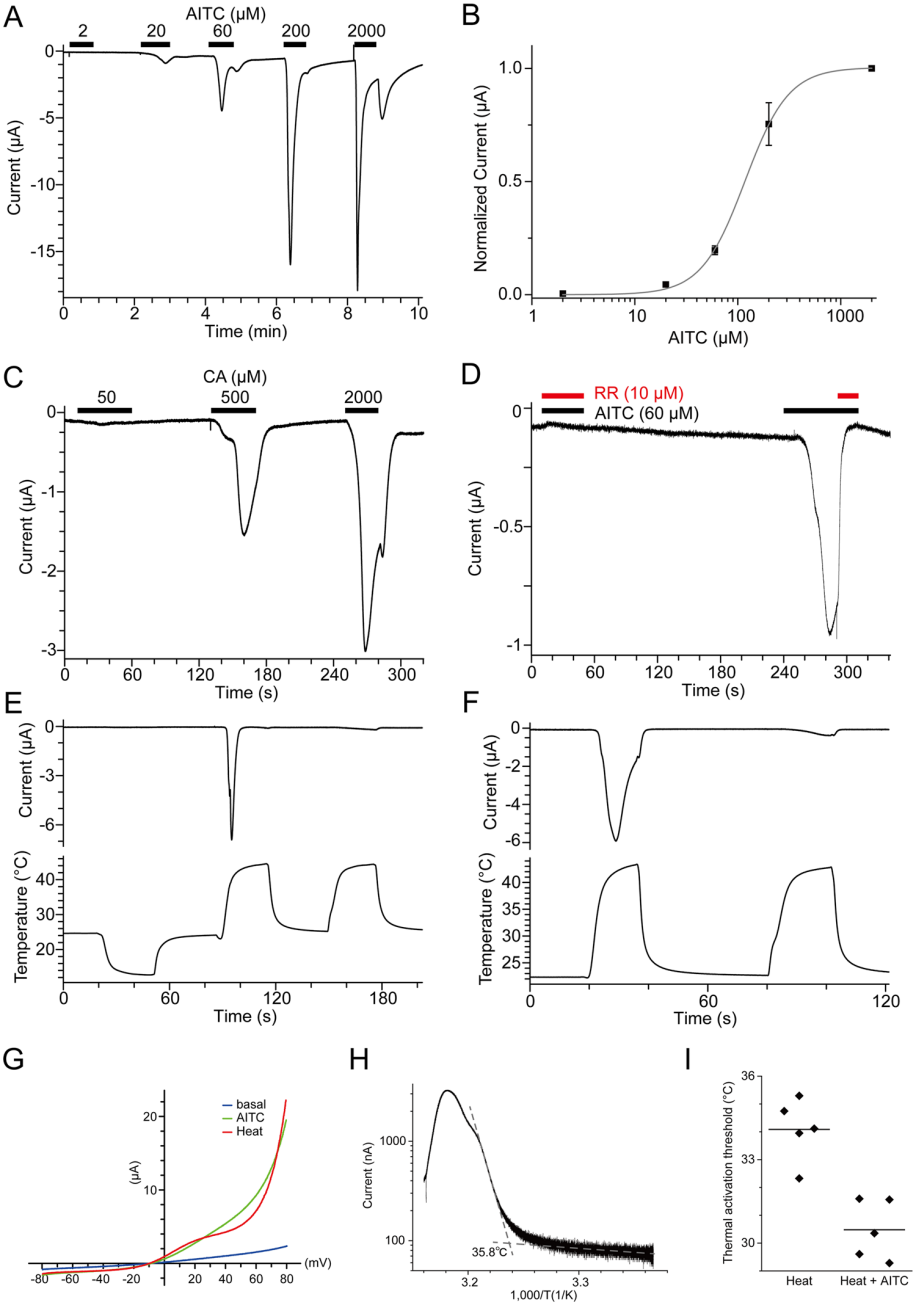
Activation of *Pp*TRPA1 by heat and chemical stimulation. (**A**, **B**) A representative current trace against AITC stimulation in *X. laevis* oocytes expressing (**A**) *Pp*TRPA1 and (**B**) its dose dependency (n = 7 or 9). **(C)** A representative current trace against cinnamaldehyde (CA) stimulation in *X. laevis* oocytes expressing *Pp*TRPA1 (n = 3). (**D**) Inhibition of AITC-evoked currents by RR in *X. laevis* oocytes expressing *Pp*TRPA1 (n = 4). Concomitant application of AITC and RR in the first stimulation completely suppressed the inward current, which was clearly observed in the second stimulation with AITC alone. (**E**, **F**) Representative current (upper) and temperature (lower) traces of thermal stimulation in *Pp*TRPA1-expressing *X. laevis* oocytes (n = 4 and 11 for E and F, respectively). (**G**) Current-voltage relationships for heat and AITC (60 μM) stimulation in *X. laevis* oocytes expressing *Pp*TRPA1. (**H**) An Arrhenius plot for the heat-evoked current in *Pp*TRPA1-expressing *X. laevis* oocytes. The data obtained from the first heat stimulation, such as that shown in panel F, was used for analysis (n = 23). (**I**) Thermal activation thresholds for *Pp*TRPA1 stimulated with or without AITC (10 μM). Thermal stimulation was applied in the presence or absence of AITC (10 μM) to *X. laevis* oocytes expressing *Pp*TRPA1, and thermal activation thresholds of heat-evoked currents were compared using the same preparation (heat alone: 34.1 ± 0.5 °C, n = 5; heat with AITC; 30.5 ± 0.5 °C, n = 5; p < 0.01 with unpaired t-test). Each dot indicates the thermal activation threshold obtained from different oocytes. Average values are shown with bars.

The thermal and chemical responses for both *Pp*TRPA1 and *Pp*TRPA basal were examined using an electrophysiological approach. The two channels were expressed separately in *Xenopus laevis* oocytes by injecting complementary RNA (cRNA) of *Pp*TRPA1 or *Pp*TRPA basal, and channel properties were examined by measuring ionic currents using the two-electrode voltage-clamp method. Allyl isothiocyanate (AITC), a well-known TRPA1 agonist, elicited clear inward currents at −60 mV in a dose-dependent manner in *X. laevis* oocytes expressing *Pp*TRPA1 (Fig. 5A). The half-maximal effective concentration (EC_50_) of AITC for *Pp*TRPA1 was 117 μM (Fig. 5B), which is similar to the value that has been observed for human TRPA1 in the *X. laevis* oocyte expression system^36^. Cinnamaldehyde (CA), another TRPA1 agonist, also evoked inward currents in *X. laevis* oocytes expressing *Pp*TRPA1 (Fig. 5C). The current evoked by AITC was completely suppressed by RR (10 μM) suggesting that the current was specifically produced by *Pp*TRPA1 (Fig. 5D).

Next, the thermal responses of *Pp*TRPA1 were examined. We first examined whether *Pp*TRPA1 was activated by cold or heat. When cold stimulation was applied to *X. laevis* oocytes expressing *Pp*TRPA1, we did not observe any response (Fig. 5E). In contrast, subsequent heat stimulation to the same oocytes evoked clear inward currents (Fig. 5E) indicating that *Pp*TRPA1 is a heat sensitive channel. We then attempted to confirm that activation of *Pp*TRPA1 by heat was not caused by the prior cold stimulation. To this end, heat-evoked currents were also observed when heat stimulation was applied to naïve *X. laevis* oocytes expressing *Pp*TRPA1 (Fig. 5F), suggesting that the prior cold stimulation is not required. Heat-evoked currents rapidly desensitized during the first heat stimulation, and only faint currents were observed in the second heat stimulation (Fig. 5E, F). This desensitization property is similar to that of heat-activated TRPA1 in several vertebrate species^26, 27^. In addition, both AITC and heat-evoked currents exhibited a clear outward rectification, which was also observed for TRPA1 from other vertebrate species (Fig. 5G). The average thermal activation threshold for *Pp*TRPA1, which was determined by Arrhenius plots, was 34.8 ± 0.5 °C (n = 23; Fig. 5H). Notably, thermal activation thresholds of *Pp*TRPA1 decreased when heat stimulation with a threshold concentration of AITC (10 μM) was applied (Fig. 5I), suggesting a synergism between thermal and chemical stimulation. In summary, *Pp*TRPA1 was activated by known TRPA1 agonists and by heat.

We also attempted to examine the channel property of *Pp*TRPA basal using the *X. laevis* oocyte expression system. However, no response was observed upon application of cold, heat, or known TRPA1 agonists (Supplementary Fig. S3).

### Effects of *PpTRPA1* knockdown on thermotactic movements of *P. pectinifera* larvae

**Figure 6:**
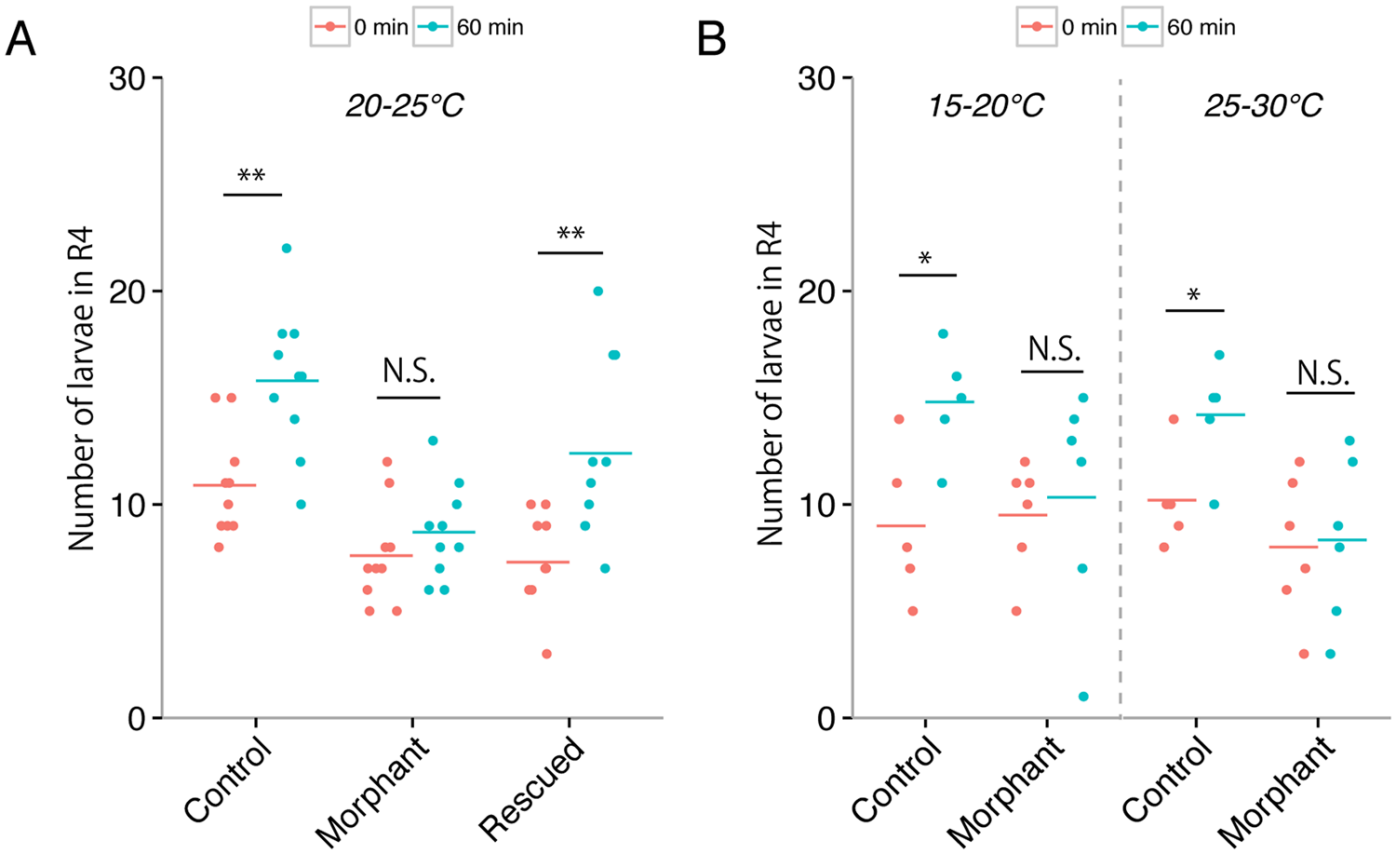
Suppression of positive thermotaxis toward warmth in *PpTRPA1* morphants. Impaired thermotaxis in *PpTRPA1* morphants (*PpTRPA1*-MO-injected larvae) in the 20–25 °C (**A**, n = 10 in each sample), 15–20 °C, and 25–30 °C gradients (**B**, morphant, n = 6; control, n = 5). Each dot shows the number of larvae that accumulated in the highest temperature range during thermotaxis assays in different thermal gradients. Bars show the mean values for each condition. *PpTRPA1*-morphants did not show any thermotactic movements in the three thermal gradients. When larvae were co-injected with *PpTRPA1* MO and synthetic *PpTRPA1* cRNA (rescued), positive thermotaxis was completely rescued (**A**). *p < 0.05 (paired t-test). **p < 0.01 (paired t-test). N. S., not significant. Control: *PpTRPA1*-CMO-injected larvae.

We tested the effects of *PpTRPA1* knockdown on the thermotactic movements of 4-day-old bipinnaria larvae using morpholino antisense oligonucleotides (MO) for the knockdown experiments and their five-mispaired control oligonucleotides (CMO) as the control. Figure 6 shows the number of larvae injected with *PpTRPA1* MO (morphant) or *PpTRPA1* CMO (control) in the highest temperature area (R4, see also the table in Fig. 2A) in each thermal gradient at 0 min and 60 min after the initiation of the accumulation assay. Compared to the start point (0 min; red dots), control larvae significantly increased in the number in the highest temperature area by the endpoint (60 min, blue dots) in the 20–25 °C gradient (Fig. 6A). In contrast, no significant differences in the number of larvae in the highest temperature area were observed in the morphant larvae between 0 min and 60 min in the 20–25 °C gradient (Supplemental Movie 5). The specificity of *PpTRPA1* MO was demonstrated in the 20–25 °C gradient by a rescue experiment with morphant larvae in which synthetic *PpTRPA1* cRNA was co-injected with *PpTRPA1* MO (Fig. 6A rescued, Supplemental Movie 6). No morphological differences were observed among the morphant, control, or rescued larvae in this experiment (Supplementary Fig. S4). In addition, in the 15–20 °C or 25–30 °C thermal gradients, *PpTRPA1* MO-injected larvae did not show any positive thermotaxis, although CMO-larvae exhibited positive thermotaxis (Fig. 6B). Together, these results suggest that *PpTRPA1* MO specifically inhibited the translation of endogenous *PpTRPA1* and suppressed positive thermotaxis in *P. pectinifera* larvae.

**Figure 7:**
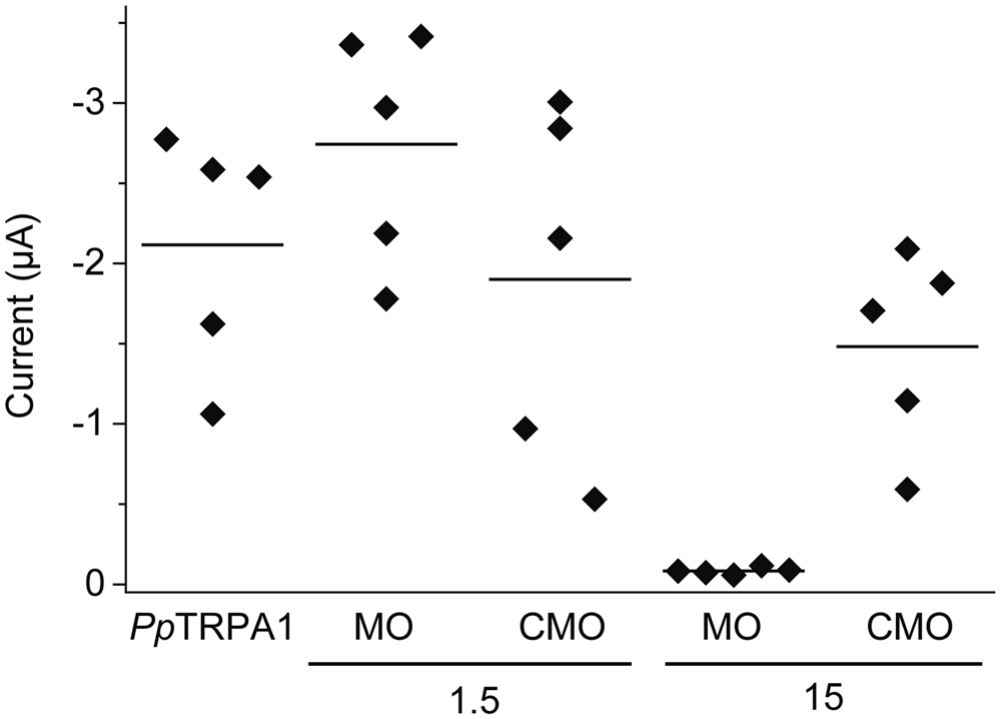
Suppression of heat-evoked currents by *PpTRPA1* MO in *X. laevis* oocytes injected with *PpTRPA1* cRNA. The amplitudes for heat-evoked currents were compared among *X. laevis* oocytes injected with *PpTRPA1* cRNA, *PpTRPA1* cRNA with MO (1.5 or 15 pmol/µL) or *PpTRPA1* cRNA with CMO (1.5 or 15 pmol/µL). Each dot indicates amplitude for the heat-evoked current obtained from each *X. laevis* oocyte. It should be noted that concomitant injection of *PpTRPA1* cRNA and *PpTRPA1* MO (15 pmol/µL) drastically suppressed heat-evoked activation. Average values are shown with bars.

We further confirmed the effect of *PpTRPA1* MO and *PpTRPA1* CMO in a heterologous expression system. *PpTRPA1* cRNA was injected into *X. laevis* oocytes with *PpTRPA1* MO (or *PpTRPA1* CMO), and current amplitudes generated by heat stimulation were compared (Fig. 7). When a higher concentration of *PpTRPA1* MO (15 pmol/µL) was injected with *PpTRPA1* cRNA into *X. laevis* oocytes, current amplitudes by heat stimulation significantly decreased. In contrast, injection of the same concentration of *PpTRPA1* CMO (15 pmol/µL) with *PpTRPA1* cRNA showed no clear effect on current amplitudes by heat stimulation. Thus, *PpTRPA1* MO specifically suppressed the translation of *PpTRPA1* in *X. laevis* oocytes.

## Discussion

Patterns of thermotactic movement are mainly divided into two types, positive and negative, in which the animals move up and down the temperature gradients, respectively^37^. Animals can seek preferable environmental temperatures by either positive or negative thermotaxis. For instance, in the fruit fly *Drosophila melanogaster*, the first instar larvae can exhibit both types of thermotaxis, depending on the environmental temperatures, and show a temperature preference of 23–29 °C^38^. In contrast, the planarians *Dugesia japonica* display only negative thermotaxis, even when the lowest temperature in the thermal gradient is 0 °C, resulting in an impairment of their movement and accumulation at approximately 0 °C^39^. In contrast, the larvae of *P. pectinifera* prefer warmth in every thermal gradient tested (Fig. 2A), thus exhibiting one-sided positive thermotaxis, even when they approach lethal temperatures (Fig. 2B, Supplemental Movie 2). We consider that the *P. pectinifera* larvae lack the avoidance of extreme heat. Avoidance behaviors, however, might not be essential for the planktonic larvae because temperatures are more stable in marine than in terrestrial environments. It is possible that the positive thermotaxis in the larvae of *P. pectinifera* could improve their feeding and/or growth patterns because phytoplankton, which serve as ‘baits’ to many zooplanktons, grow well at high temperatures^40^.

In the present study, we characterized the transient receptor potential ankyrin (TRPA) from the *P. pectinifera* larvae and demonstrated that *Pp*TRPA1 acts as a thermosensor for positive thermotaxis. This was evident from the results of an electrophysiological analysis in a heterologous expression system using *X. laevis* oocytes (Fig. 5), and *in vivo* analyses using MOs, followed by the rescue experiments in the *P. pectinifera* larvae (Fig. 6). To the best of our knowledge, this is the first report that focuses on the relationship between thermo TRP channels and thermotaxis in marine planktonic larvae. Here, we discuss our findings from different viewpoints concerning the sensory neuronal cells, behavioral diversity triggered by TRPA1 channels, channel activity of *Pp*TRPA1, practical situation on the operation of *Pp*TRPA1 in habitat, and another type of *Pp*TRPA molecule (*Pp*TRPA-basal).

Several studies have reported the expression of TRPA1 channels in the sensory neurons of vertebrates^41–44^ and invertebrates^45–47^. We assumed that *PpTRPA1* was also expressed in the sensory neurons of the *P. pectinifera* larvae. This notion was well supported by fluorescent *in situ* hybridization, which led to the detection of a small number of *PpTRPA1*-expressing cells with scattered distribution in the ciliary band (Fig. 4). In the *P. pectinifera* larvae, the ciliary band is equipped with an elaborate nervous system, in which the columnar epithelial cells coexist with several kinds of neuronal cells. Similar to other marine invertebrate larvae, the neuronal cells of the starfish larvae are thought to regulate the ciliary movement of columnar-epithelial cells^48, 49^, and are consequently specialized for their swimming and feeding behaviors^50, 51^. The *PpTRPA1*-expressing cells might trigger a signal transduction downstream of the neuronal circuit immediately after sensing heat. To the best of our knowledge, no other study describes the sensory neurons showing a distribution characteristic of the starfish larvae.

The divergent relationships between TRPA1 channel functions and behavioral outputs in various animals are of particular interest to physiologists. For example, in several vertebrate species, the TRPA1 channels function as receptors for noxious thermal and chemical stimuli, and are therefore involved in avoidance behavior^21, 24, 26, 27, 29^. However, in snake species with pit organs, TRPA1 has been reported to play a role in sensing infrared radiation, which is not always linked to avoidance behaviors^52^. These above-mentioned divergent usages might be due to different signal transduction processes triggered by thermal stimuli in the neural circuits via TRPA1 channels, thereby leading to different behavioral outcomes. In the present study, we found that the *Pp*TRPA1 channel is likely responsible for the preference of warmth in the larvae of the echinoderm, *P. pectinifera*.

The thermal activation threshold for *Pp*TRPA1 (approximately 35 °C) did not correspond to the range of temperatures at which *P. pectinifera* larvae demonstrated positive thermotaxis (15–33 °C). Similar inconsistencies have been reported in another study on the relationship between temperature-dependent sex determination and TRP vanilloid 4 (TRPV4) thermal sensitivity in alligators^53^. These inconsistencies might be explained by the reasons outlined below. Firstly, a modulator might play a role in deceasing the thermal activation threshold of *Pp*TRPA1 in the *P. pectinifera* larvae, but such a modulator is absent in *X. laevis* oocytes, which were used for the ectopic expression experiments. Indeed, the thermal activation threshold of *Pp*TRPA1 decreased by several degrees Celsius in the presence of a threshold concentration of allyl isothiocyanate (AITC) in *X. laevis* oocytes (Fig. 5I). If the endogenous activators of TRPA1 exist in the *P. pectinifera* larvae, TRPA1 could be activated by the temperature ranges used in the thermotaxis assays. Secondly, the differences between the plasma membrane compositions of *P. pectinifera* larvae and *X. laevis* oocytes might influence the thermal activation threshold of *Pp*TRPA1.

Another significant problem that should be paid attention to in the present study is concerned with the practical implication of *Pp*TRPA1 as a thermal receptor in the habitat of the *P. pectinifera* larvae. Our experimental condition, in which the steady-state thermal gradient formed at five degrees within a distance of 4.5 cm (Fig. 1), would not reflect any ambient spatiotemporal scales of the thermal conditions in a habitat. Furthermore, the water dynamics of a habitat would frequently be the leading cause of disturbance in the constant conditions of a thermal gradient. However, it could not be denied that the thermal gradient might form in several situations such as (i) when a boundary face encounters the high and low temperatures of water bodies^54^ and (ii) when great temperature extremes are caused over relatively short distances in a microhabitat, such as tidepool^55^. Nevertheless, at present, we were unable to determine any solutions for this problem, but we considered that *Pp*TRPA1 is essentially utilized for the progression of larval stages. The *Pp*TRPA1 genes have been maintained not only in a closely related species, *P miniata*, but also in distantly related species, such as vertebrates or insects, without showing any acceleration of amino acid substitutions, suggesting the conserved mode of evolution (Fig. 3). Therefore, *P. pectinifera* is widely distributed around the sea coast of eastern Asia.

In addition to TRPA1, several other channels belonging to the TRPA subfamily serve as thermal sensors in invertebrate species^23, 28, 30^. Our examination of the thermal and chemical sensitivity of *Pp*TRPA-basal in the *X. laevis* oocyte expression system did not show any response (Supplementary Fig. S3). This might be due to the lack of membrane expression of *Pp*TRPA-basal in *X. laevis* oocytes. Alternatively, we might have failed to identify the proper stimuli that activate *Pp*TRPA1-basal. However, it is likely that *Pp*TRPA-basal also plays certain physiological roles in starfishes, since it has been phylogenetically retained with an adaptive significance as in the case of *Pp*TRPA1 (Fig. 3B, see above paragraph in “Discussion”). In addition, *Pp*TRPA-basal retained the typical domain structures, such as ankyrin repeats and six transmembrane domains, which are required to form an ion permeation pathway (Fig. 3A). Interestingly, *Pp*TRPA-basal was spatially confined to the posterior region of the digestive tract in the bipinnaria larval stage (Fig. 4). In mammals, the physiological roles of TRPA1 in the digestive tract have been proposed^56^. The molecular and cellular investigation of *Pp*TRPA-basal would provide meaningful insights into the understanding of digestive functions in marine planktonic larvae.

## Materials and Methods

### *P. pectinifera* larvae and dejellied egg preparation

Adult *P. pectinifera* were collected from the Sagami Bay, Kanagawa Prefecture and the Mutsu Bay, Aomori Prefecture, Japan, and maintained in natural or artificial seawater (ASW; Marineart SF-1, Tomita Pharmaceutical, Japan) at 15 °C. Larvae were obtained as previously described^57^. In brief, immature eggs were treated with 1-methyladenine^58^ (1-MA; Sigma) and then fertilized with diluted dry sperm. Embryos were reared for four days in artificial seawater (Jamarin U; Jamarin Laboratory, Japan) at 20 °C until they reached the bipinnaria larval stage.

For the injection experiments with morpholino antisense oligonucleotides and synthetic *Pp*TRPA1 complementary RNA (cRNA), dejellied eggs were obtained as follows: ovaries were cut into small pieces in Ca^2+^-free seawater (CFSW, Jamarin Laboratory, Japan), stored at room temperature (RT) for 10 min, and washed three times with Jamarin U at RT. Eggs were then treated with low pH (pH 4) seawater at RT for less than 1 min and washed three times with Jamarin U at RT.

### Chemicals

In each stock solution, allyl isothiocyanate (Kanto Chemical), cinnamaldehyde (Wako), and carvacrol (Wako) was dissolved at a concentration of 2 M into dimethyl sulfoxide, respectively. Ruthenium red (Sigma) was dissolved at a concentration of 10 mM into water. These stock solutions were used for thermotaxis assay and channel property analyses.

### Thermotaxis assay for *P. pectinifera* larvae

Thermotaxis was examined using the accumulation assay system shown in Fig. 1. The device consisted of two parts: a thermal gradient stage and a chamber filled with Jamarin U containing larvae. To create the thermal gradient, one side of the stage was heated with hot water (25–37 °C), and the other side was cooled with cold water (8–20 °C) ﻿(Fig. 1C)﻿ depending on the temperature range used for that particular assay. Gradients formed in the chamber within 3 min were maintained during the course of the assays (Fig. 1B). Five thermosensors were evenly placed beneath the chamber, and the temperature reading by each thermosensor was monitored. In addition, thermographic images of the chambers were recorded with a Thermo Tracer thermal imager (TH9100MV/WV, NECSAN-EI, Japan). Approximately 50 bipinnaria larvae (4 days post-fertilization, Fig. 1A) were uniformly placed into a chamber with a mouth pipette at RT, and the chamber was then placed on the thermal stage. In some experiments (Fig. 2D), larvae were washed twice with Jamarin U containing 20 µM of ruthenium red prior to thermotaxis assay, and placed in the chamber as described above. Time-lapse images were taken every 20 sec (D300S (Nikon) with an AF-S DX Micro NIKKOR 85 mm f/3.5 G ED VR lens (Nikon) controlled by a Camera Control Pro 2 (Canon)) to record movements of the larvae within the chamber. All images were processed using Image J (National Institutes of Health, Bethesda, Maryland, USA, http://imagej.nih.gov/ij) to generate time-lapse videos. The number of larvae in each area was counted manually or with the particle counting tool in Image J.

### Search for TRPA homologs in starfish

Scaffolds of the *P. miniata* genome were obtained from an NCBI BioProject (GCA_000285935.1)^59^. We used the software AUGUSTUS with a human model to predict protein coding sequences in these scaffolds^60^. We additionally used the software HMMER to identify TRPA homologs in predicted genes from *P. miniata* ^34^. The known TRPA protein sequences were used to construct an HMMER profile and are listed in Supplemental Table 1. We identified two sequences that possibly code for a TRPA1-like protein in the genome of *P. miniata*. To confirm the existence of two TRPA1-like copies in *P. pectinifera*, we performed RNA-seq. Total RNA was extracted with a FastPure RNA Kit (TAKARA, Japan) following the manufacturer’s instructions for early mixed embryos, including from the fertilized egg to mid-gastrula stage, late gastrulae stage, and bipinnaria larval stage. Library construction, RNA sequencing, and de novo assembly were performed as previously described^61^ by the Beijing Genomics Institute (BGI)-Shenzhen, Shenzhen, China (http://www.genomics.cn). We identified two copies of TRPA, but we could not find a third copy of TRPA in the transcriptome of *P. pectinifera* larvae with the HMMER search.

### Phylogenetic analysis of *PpTRPA1* and *PpTRPAbasal*

We collected known protein sequences of TRPA and aligned them with the two TRPA-like genes identified in *P. pectinifera* and *P. miniata*. We used CLUSTAL W2^62^ to align only the transmembrane regions, which were identified by a Pfam search^35^. The accession numbers of the sequences used here are listed in Supplemental Table 2. We reconstructed a phylogenetic tree with the NJ method using p-distance (partial deletion, amino acids used for pairwise comparison were from 100 to 291 residues and 225.2 residues on average)^63^. As a result, one copy clustered in the TRPA1 clade while the other copy clustered in the TRPA basal clade^19, 37^. We named the copy of *P. pectinifera* in the TRPA1 clade as “*PpTRPA1*” and the copy of *P. pectinifera* in TRPA basal clade as “*PpTRPA basal*”.

### Gene cloning and gene structures

To isolate the *PpTRPA1* gene, RT-PCR was carried out using the primer set (forward 5′-GCTAGCGTTTAAACTTAAGCTTACTGTA**ATG**GAGGGCGACGAGC-3′ and reverse 5′-GCCCTCTAGACTCGAGCGGCCGC **TCA**GGCTGTGTTGGAGCCATCGTGTTCGC-3′; underlined nucleotides are additional sequences that contained restriction enzyme recognition sites for cloning; initiation and stop codons are shown with bold letters) and cDNA synthesized with total RNA from the gastrulae of *P. pectinifera* using ReverTra Ace (TOYOBO). The amplified PCR fragment containing a full-length coding sequence of *PpTRPA1* was cloned into pcDNA3.1 using *Hind*III and *Not*I. Then, the *PpTRPA1* coding sequence was subcloned into the pOX + vector.

The full-length coding sequence of *PpTRPA basal* was obtained by RT-PCR using the primer set (forward 5′-**ATG**AACCCATCAGGAAA-3′ and reverse 5′-**TTA**CTCTCCCACTTC-3′) and cDNA synthesized with total RNA extracted from the bipinnaria larvae. Then, the amplified PCR fragment was cloned into the pOX + vector. The full length coding sequence for *PpTRPA basal* (1398 codons) was a little shorter than that of *PpTRPA basal* that was predicted by the RNA-seq analysis (1406 codons). We used *PpTRPA basal* with 1398 amino acids for phylogenetic reconstruction and electrophysiological experiments. The domain structures for *PpTRPA1* or *PpTRPA basal* were predicted with Pfam^35^.

### Whole-mount *in situ* hybridization

Whole-mount *in situ* hybridization (WISH) was carried out as previously described with the modification that 4-day bipinnaria were treated with proteinase K for 2 h for experiments using the *PpTRPA1* probe^64^. To detect fluorescent signals, an HNPP Fluorescent Detection Set (Roche) was used. Coronal plane stack images were obtained by using the confocal leaser scanning microscope system (Fluoview, FV300, Olympus, Tokyo, Japan) and z-stack images were generated using Image J software. The probes for *PpTRPA1* and *PpTRPA basal* included from 1 bp to 1812 bp and from 2663 bp to 4170 bp of the open reading frame sequences, respectively.

### Quantitative polymerase chain reaction (qPCR)

QPCR was performed as previously described with some modifications^65^. Total RNA from 200 or 800 bipinnaria larvae of *P. pectinifera* was isolated using a NucleoSpin® RNA Kit (MACHEREY-NAGEL), and reverse transcription was performed using a High-Capacity cDNA Reverse Transcription Kit (Applied Biosystems) according to the manufacturer’s protocol. A KAPA SYBR® FAST qPCR Master Mix (2X) ABI Prism^TM^ Kit (KAPA Biosystems) was used for PCR reactions carried out with a StepOnePlus Real-Time PCR System (Applied Biosystems). Primer pairs used for PCR reactions were as follows: *PpTRPA1*, 5′-CCCCGTGTGCGTTAACTACT-3′ and 5′-CGTGGCTGGATTTAAGGTGT-3′; *PpTRPA basal*, 5′-TTGACGGTTTTGTTGGATC-3′ and 5′-CGTGTTGGAATCCTCGTCT-3′. PCR reactions for negative controls were carried out with a reverse transcribed mixture lacking enzyme.

### Two-electrode voltage clamp method

*Pp*TRPA1 and *Pp*TRPA basal were heterologously expressed in *X. laevis* oocytes, and ionic currents were recorded using a previously described two-electrode voltage-clamp method^66^. Complementary RNA (cRNA) was synthesized using linearized pOX + vectors containing *PpTRPA1* (or *PpTRPA1 basal)* with an mMESSAGE MACHINE SP6 Transcription Kit (Ambion) according to the manufacturer’s instructions. Mature *X. laevis* females were purchased from Hamamatsu Seibutsu Kyozai (Hamamatsu, Japan) and reared at ~18 °C. Oocytes were surgically excised from anesthetized *X. laevis*, and follicular membranes were enzymatically removed from oocytes as previously described^66^. Fifty nanoliters of cRNA (150 and 100 ng/µL for *PpTRPA1* and *PpTRPA basal*, respectively) was injected into defolliculated oocytes, and ionic currents were recorded 3–6 days post-injection using an OC-725C amplifier (Warner Instruments) with a 1 kHz low-pass filter and digitized at 5 kHz with a Digidata® 1440 System (Axon Instruments). Oocytes were voltage-clamped at –60 mV. Each of stock solutions of chemicals was further dissolved into an ND96 bath solution (96 mM NaCl, 2 mM KCl, 1.8 mM CaCl_2_, 1 mM MgCl_2_, and 5 mM 2-[4-(2-Hydroxyethyl)-1-piperazinyl] ethanesulfonic acid (HEPES), pH 7.4) and applied to the oocytes by perfusion. Heated (or cooled) ND96 was applied by perfusion for heat (or cold) stimulation. Temperature was monitored with a thermistor located just beside the oocytes using a temperature controller (TC-344B, Warner Instruments). Thermal activation thresholds for *Pp*TRPA1 were determined with an Arrhenius plot that was created using the Clampfit 10.4 (Molecular Devices) and Origin 9 J software (OriginLab). The EC_50_ value was determined using the Origin 9 J. Current amplitudes were compared among *X. laevis* oocytes injected with 50 nL of *PpTRPA* cRNA (150 ng/µL), *PpTRPA1* cRNA (150 ng/µL) with MO (1.5 or 15 pmol/µL), or *PpTRPA1* cRNA (150 ng/µL) with CMO (1.5 or 15 pmol/µL). *X. laevis* oocytes prepared from a single frog were used and currents amplitudes against heat stimulation were measured after 4 days post injection for all conditions. A current-voltage relationship was obtained by applying ramp pulse (from −80 to + 80 mV during 0.5 sec). All procedures involving the care and use of animals were approved by the committees for animal experimentation of the National Institute for Physiological Sciences (Japan).

### Microinjection of morpholino antisense oligonucleotides and cRNA

Dejellied eggs were arrayed in a row on a plastic culture dish coated with 1% protamine sulfate (Wako Chemicals, Japan). The morpholino antisense oligonucleotide (MO; Gene Tools, USA) sequences of the first-Met blocking and five-mispaired control morpholino oligonucleotides (CMOs) were as follows: *PpTRPA1*-MO, 5′-TACGCTCGTCGCCCTCCATTACAGT-3′; *PpTRPA1*-CMO, 5′-TACaCTCaTCGaCCTCaATTACAaT-3′. Lowercase letters in the CMO sequence indicate mispaired nucleotides. To construct cRNA for the rescue experiments, silent mutations were introduced at the MO-binding sites in *PpTRPA1*. The QuickChange Site-Directed Mutagenesis Kit (Agilent Technologies, USA) was used with the primers 5′-GAATTCTGCAGATAAACTTAAGCTTACgGTcATGGAaGGaGAtGAaCGgACGCCTGTGCCGCGGG-3′ (lowercase letters indicate the introduced silent mutations) and 5′-CCCGCGGCACAGGCGTCCGTTCATCTCCTTCCATGACCGTAAGCTTAAGTTTATCTGCAGAATTC-3′ according to the manufacturer’s instruction to create the recombinant plasmid vector pOX-*PpTRPA1*. To synthesize the 5′-capped cRNA, pOX-*PpTRPA1* was linearized with *Mlu*I, and then used as a template for the *in vitro* synthesis of the 5′-capped cRNA using an mMESSAGE mMACHINE SP6 kit (Ambion, USA). MO and CMO were used at concentrations of 168 μM in 5 μg/µL of dextran-tetramethylrhodamine (10,000 MW, lysine fixable; Invitrogen, USA). For the rescue experiments, a 10-pl mixture comprising *PpTRPA1* cRNA (with silent mutations), MO, and dextran-tetramethylrhodamine was microinjected into the discharged eggs using a micromanipulator (Narishige, Japan) and FemtoJet Microinjector (Eppendorf, Germany). Subsequently, the final concentrations of *PpTRPA1* cRNA (with silent mutations), MO, and rhodamine were 2 µg/µL, 168 µM, and 5 µg/µL, respectively. The MO- or CMO-injected eggs were treated with 1-methyladenine (1-MA) for 15 min at RT for maturation, fertilized with diluted dry sperm, and allowed to develop until four days post-fertilization for conducting the accumulation assays as described above.

## Electronic supplementary material

Supplementary information

Movie 1

Movie 2

Movie 3

Movie 4

Movie 5

Movie 6

## References

[1] Pechenik, J. A. On the advantages and disadvantages of larval stages in benthic marine invertebrate life cycles. *Mar Ecol Prog Ser* **177**, 269–297, doi:10.3354/meps177269 (1999).

[2] Flynn KJ (2012). Changes in pH at the exterior surface of plankton with ocean acidification. Nature Clim. Change.

[3] Balzano S, Sarno D, Kooistra WHCF (2010). Effects of salinity on the growth rate and morphology of ten Skeletonema strains. Journal of Plankton Research.

[4] Gismervik I (2006). Top-down impact by copepods on ciliate numbers and persistence depends on copepod and ciliate species composition. Journal of Plankton Research.

[5] Sohn MH, Lim S, Seo KW, Lee SJ (2013). Effect of ambient medium viscosity on the motility and flagella motion of Prorocentrum minimum (Dinophyceae). Journal of Plankton Research.

[6] Jekely G (2008). Mechanism of phototaxis in marine zooplankton. Nature.

[7] Chen CY, Folt CL (2002). Ecophysiological responses to warming events by two sympatric zooplankton species. Journal of Plankton Research.

[8] Winder M (2012). Spring phenological responses of marine and freshwater plankton to changing temperature and light conditions. Marine Biology.

[9] Mah, C. L. & Blake, D. B. Global diversity and phylogeny of the Asteroidea (Echinodermata). *PLoS One* **7**, e35644, doi:10.1371/journal.pone.0035644 (2012). 10.1371/journal.pone.0035644 PMC3338738 22563389

[10] Matsuoka N, Asano H (2003). Genetic variation in northern Japanese populations of the starfish Asterina pectinifera. Zoolog Sci.

[11] Lee AR (2012). Cloning, Heterologous Expression, and Enzymatic Characterization of Cathepsin L from Starfish (Asterina pectinifera). Bioscience, Biotechnology, and Biochemistry.

[12] Satoh SK (2013). The tension at the top of the animal pole decreases during meiotic cell division. PLoS One.

[13] Caterina MJ (1997). The capsaicin receptor: a heat-activated ion channel in the pain pathway. Nature.

[14] Bandell M, Macpherson LJ, Patapoutian A (2007). From chills to chilis: mechanisms for thermosensation and chemesthesis via thermoTRPs. Curr Opin Neurobiol.

[15] Dhaka A, Viswanath V, Patapoutian A (2006). Trp ion channels and temperature sensation. Annu Rev Neurosci.

[16] Patapoutian A, Peier AM, Story GM, Viswanath V (2003). ThermoTRP channels and beyond: mechanisms of temperature sensation. Nat Rev Neurosci.

[17] Liao M, Cao E, Julius D, Cheng Y (2013). Structure of the TRPV1 ion channel determined by electron cryo-microscopy. Nature.

[18] Paulsen CE, Armache JP, Gao Y, Cheng Y, Julius D (2015). Structure of the TRPA1 ion channel suggests regulatory mechanisms. Nature.

[19] Kang K (2010). Analysis of Drosophila TRPA1 reveals an ancient origin for human chemical nociception. Nature.

[20] Saito S, Shingai R (2006). Evolution of thermoTRP ion channel homologs in vertebrates. Physiol Genomics.

[21] Saito S, Tominaga M (2015). Functional diversity and evolutionary dynamics of thermoTRP channels. Cell Calcium.

[22] Kadowaki T (2015). Evolutionary dynamics of metazoan TRP channels. Pflugers Arch.

[23] Lee Y (2005). Pyrexia is a new thermal transient receptor potential channel endowing tolerance to high temperatures in Drosophila melanogaster. Nat Genet.

[24] Prober DA (2008). Zebrafish TRPA1 channels are required for chemosensation but not for thermosensation or mechanosensory hair cell function. J Neurosci.

[25] Rosenzweig M (2005). The Drosophila ortholog of vertebrate TRPA1 regulates thermotaxis. Genes Dev.

[26] Saito S (2014). Heat and noxious chemical sensor, chicken TRPA1, as a target of bird repellents and identification of its structural determinants by multispecies functional comparison. Mol Biol Evol.

[27] Saito S (2012). Analysis of transient receptor potential ankyrin 1 (TRPA1) in frogs and lizards illuminates both nociceptive heat and chemical sensitivities and coexpression with TRP vanilloid 1 (TRPV1) in ancestral vertebrates. J Biol Chem.

[28] Sokabe T, Tsujiuchi S, Kadowaki T, Tominaga M (2008). Drosophila painless is a Ca2 + -requiring channel activated by noxious heat. J Neurosci.

[29] Story GM (2003). ANKTM1, a TRP-like channel expressed in nociceptive neurons, is activated by cold temperatures. Cell.

[30] Tracey WD, Wilson RI, Laurent G, Benzer S (2003). painless, a Drosophila gene essential for nociception. Cell.

[31] Viswanath V (2003). Opposite thermosensor in fruitfly and mouse. Nature.

[32] Corfas, R. A. & Vosshall, L. B. The cation channel TRPA1 tunes mosquito thermotaxis to host temperatures. *Elife* **4**, 10.7554/eLife.11750 (2015). 10.7554/eLife.11750 PMC4718722 26670734

[33] Nilius B, Owsianik G (2011). The transient receptor potential family of ion channels. Genome Biol.

[34] Johnson LS, Eddy SR, Portugaly E (2010). Hidden Markov model speed heuristic and iterative HMM search procedure. BMC Bioinformatics.

[35] Finn RD (2014). Pfam: the protein families database. Nucleic Acids Res.

[36] Cordero-Morales JF, Gracheva EO, Julius D (2011). Cytoplasmic ankyrin repeats of transient receptor potential A1 (TRPA1) dictate sensitivity to thermal and chemical stimuli. Proc Natl Acad Sci USA.

[37] Garrity PA, Goodman MB, Samuel AD, Sengupta P (2010). Running hot and cold: behavioral strategies, neural circuits, and the molecular machinery for thermotaxis in C. elegans and Drosophila. Genes Dev.

[38] Luo L (2010). Navigational decision making in Drosophila thermotaxis. J Neurosci.

[39] Inoue T, Yamashita T, Agata K (2014). Thermosensory signaling by TRPM is processed by brain serotonergic neurons to produce planarian thermotaxis. J Neurosci.

[40] Kiefer, D. A. & Cullen, J. J. Phytoplankton growth and light absorption as regulated by light, temperature, and nutrients. *1991* **10**, 10, 10.3402/polar.v10i1.6735 (1991).

[41] Kim YS (2010). Expression of transient receptor potential ankyrin 1 (TRPA1) in the rat trigeminal sensory afferents and spinal dorsal horn. J Comp Neurol.

[42] Yonemitsu T (2013). TRPA1 detects environmental chemicals and induces avoidance behavior and arousal from sleep. Sci Rep.

[43] Bautista DM (2005). Pungent products from garlic activate the sensory ion channel TRPA1. Proc Natl Acad Sci USA.

[44] Caspani O, Zurborg S, Labuz D, Heppenstall PA (2009). The contribution of TRPM8 and TRPA1 channels to cold allodynia and neuropathic pain. PLoS One.

[45] Kang K (2012). Modulation of TRPA1 thermal sensitivity enables sensory discrimination in Drosophila. Nature.

[46] Lee Y (2013). Contribution of Drosophila TRPA1-expressing neurons to circadian locomotor activity patterns. PLoS One.

[47] Luo J, Shen WL, Montell C (2017). TRPA1 mediates sensation of the rate of temperature change in Drosophila larvae. Nat Neurosci.

[48] Mackie GO, Spencer AN, Strathmann R (1969). Electrical activity associated with ciliary reversal in an Echinoderm larva. Nature.

[49] Satterlie RA, Cameron RA (1985). Electrical activity at metamorphosis in larvae of the sea urchin Lytechinus pictus (Echinoidea: Echinodermata). J. Exp. Zool..

[50] Stephens RE (2008). Ciliogenesis, ciliary function, and selective isolation. ACS Chem Biol.

[51] Strathmann RR (2007). Time and extent of ciliary response to particles in a non-filtering feeding mechanism. Biol Bull.

[52] Gracheva EO (2010). Molecular basis of infrared detection by snakes. Nature.

[53] Yatsu R (2015). TRPV4 associates environmental temperature and sex determination in the American alligator. Sci Rep.

[54] Cheriton OM, McManus MA, Stacey MT, Steinbuck JV (2009). Physical and biological controls on the maintenance and dissipation of a thin phytoplankton layer. Mar Ecol Prog Ser.

[55] Nakamura R (1976). Temperature and the Vertical Distribution of Two Tidepool Fishes (Oligocottus maculosus, O. snyderi). Copeia.

[56] Holzer P (2011). Transient receptor potential (TRP) channels as drug targets for diseases of the digestive system. Pharmacol Therapeut.

[57] Dan-Sohkawa M, Satoh N (1978). Studies on dwarf larvae developed from isolated blastomeres of the starfish. Asterina pectinifera. J Embryol Exp Morphol.

[58] Kanatani H (1969). Induction of spawning and oocyte maturation by L-methyl-adenine in starfishes. Exp Cell Res.

[59] Barrett T (2012). BioProject and BioSample databases at NCBI: facilitating capture and organization of metadata. Nucleic Acids Res.

[60] Stanke M, Steinkamp R, Waack S, Morgenstern B (2004). AUGUSTUS: a web server for gene finding in eukaryotes. Nucleic Acids Res.

[61] Zhang X (2013). mRNA-seq analysis of the Gossypium arboreum transcriptome reveals tissue selective signaling in response to water stress during seedling stage. PLoS One.

[62] Larkin MA (2007). Clustal W and Clustal X version 2.0. Bioinformatics.

[63] Saitou N, Nei M (1987). The neighbor-joining method: a new method for reconstructing phylogenetic trees. Mol Biol Evol.

[64] Kawai N, Kuraishi R, Kaneko H (2016). Wnt, Frizzled, and sFRP gene expression patterns during gastrulation in the starfish Patiria (Asterina) pectinifera. Gene Expr Patterns.

[65] Ransick A (2004). Detection of mRNA by *in situ* hybridization and RT-PCR. Methods Cell Biol.

[66] Saito S, Fukuta N, Shingai R, Tominaga M (2011). Evolution of vertebrate transient receptor potential vanilloid 3 channels: opposite temperature sensitivity between mammals and western clawed frogs. PLoS Genet.

