# Supplementary material for: Characterization of TRPA channels in the starfish Patiria pectinifera: involvement of thermally activated TRPA1 in thermotaxis in marine planktonic larvae
Source: Sci Rep. 2017 May 19;7:2173. doi: 10.1038/s41598-017-02171-8 (PMC5438368; doi:10.1038/s41598-017-02171-8)
Supplement: Supplementary file 1 — Supplementary information [file 41598_2017_2171_MOESM1_ESM.pdf]

Supplementary information

Characterization of TRPA channels in the starfish *Patiria pectinifera*: involvement of thermally activated TRPA1 in thermotaxis in marine planktonic larvae

Shigeru Saito[1, 2‡], Gen Hamanaka[3‡], Narudo Kawai[4], Ryohei Furukawa[5], Jun Gojobori[6], Makoto Tominaga[1, 2], Hiroyuki Kaneko[4]*, and Yoko Satta[6]*

Supplementary table

Supplementary Table 1. The list of accession numbers of genes used for profile construction in hmmer.

ADG84993	XP_001493514
ADG84994	XP_001926150
AAP76197	XP_581588
XP_001843992	AEL30802
ACC86138	AEL30803
EFA01253	XP_003408268
XP_001944501	XP_001378427
XP_002121983	XP_003205160
NP_997491	XP_418294
EDL14332	XP_002197858
XP_003509521	ADD82928
NP_001185699	ADD82932
EHB06406	ADD82929
XP_001083172	ADD82930
EHH64231	XP_003219691
XP_519806	NP_001121434
NP_015628	CBN81913
XP_002819221	XP_003443514
XP_003274822	ACI26673
XP_002759054	CAM46983
XP_544123	XP_002741542
XP_002922845	XP_003388069

Supplementary Table 2. List of genes used in a tree of Figure 3

Sequence name appears in the tree	species	Accsession number
Giant panda	Ailuropoda melanoleuca	XP_002922845
African malaria mosquito	Anopheles gambiae	ACC86138
C_briggsae	Caenorhabditis briggsae	CAP26216
C_elegans	Caenorhabditis elegans	NP_502249.3
Marmoset	Callithrix jacchus	XP_002759054
Dog	Canis lupus familiaris	XP_544123
Bat	Carollia brevicauda	AEL30802.1
Guinea pig	Cavia porcellus	NP_001185699
Sea squirt	Ciona intestinalis	XP_002121983
Boa	Corallus hortulanus	ADD82932
Rattlesnake	Crotalus atrox	ADD82930
Southern house mosquito	Culex quinquefasciatus	XP_001843992
Fruit fly_painless	Drosophila melanogaster	NP_611979
Fruit fly_pyrexia	Drosophila melanogaster	NP_612015
Fruit fly_water_witch	Drosophila melanogaster	NP_731193
Fruit fly_TRPA1	Drosophila melanogaster	NP_648263
Zebrafish1a	Danio rerio	XP_009296845
Zebrafish1b	Danio rerio	XP_009295606
Vampire bat	Desmodus rotundus	AEL30803
Seabass	Dicentrarchus labrax	CBN81913
D_mojavensis	Drosophila mojavensis	XP_002007415
D_virillis	Drosophila virilis	ADG84994
Horse	Equus caballus	XP_001493514
Chicken	Gallus gallus	BAO51998
Naked mole-rat	Heterocephalus glaber	EHB06406
Human	Homo sapiens	NP_015628
Elephant	Loxodonta africana	XP_003408268
Crab-eating macaque	Macaca fascicularis	EHH64231
Rhesus monkey	Macaca mulatta	XP_005563579
Opossum	Monodelphis domestica	XP_001378427

Mouse	Mus musculus	NP_808449
Sea anemone1	Nematostella vectensis	XP_001625280.1
Sea anemone2	Nematostella vectensis	XP_001630764.1
Sea anemone3	Nematostella vectensis	XP_001631352.1
Sea anemone4	Nematostella vectensis	XP_001633166.1
Sea anemone5	Nematostella vectensis	XP_001639740.1
Sea anemone6	Nematostella vectensis	XP_001641230.1
Sea anemone7	Nematostella vectensis	XP_001619454.1
Gibbon	Nomascus leucogenys	XP_003274822.1
Nile tilapia	Oreochromis niloticus	XP_003443514
Chimpanzee	Pan troglodytes	XP_519806
Rat snake	Pantherophis obsoletus lindheimeri	ADD82929
Orangutan	Pongo abelii	XP_002819221
Python	Python regius	ADD82928
Rat	Rattus norvegicus	NP_997491
Pig	Sus scrofa	XP_006825038
Zebra finch	Taeniopygia guttata	XP_013852128
Torafugu	Takifugu rubripes	XP_002197858
Red flour beetle	Tribolium castaneum	EFA01253
Tropical clawed frog	Xenopus tropicalis	NP_001121434

Supplementary figure

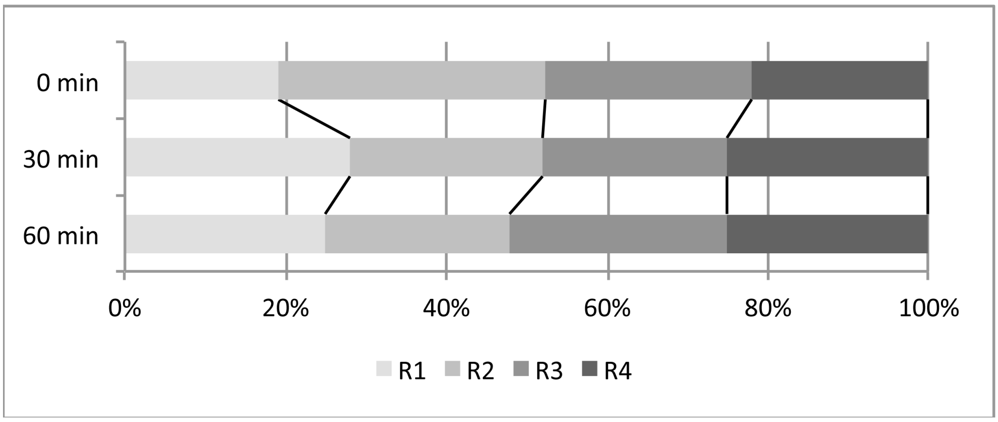

Supplementary Fig. S1. Thermotactic behavior of starfish larvae at constant temperature. Approximately 50 larvae were added to a chamber, and the number of larvae was counted as in the same way as in Fig. 2. The proportions of the number of larvae in each area remained relatively constant over time (n = 3).

***PpTRPA1* sense probe**

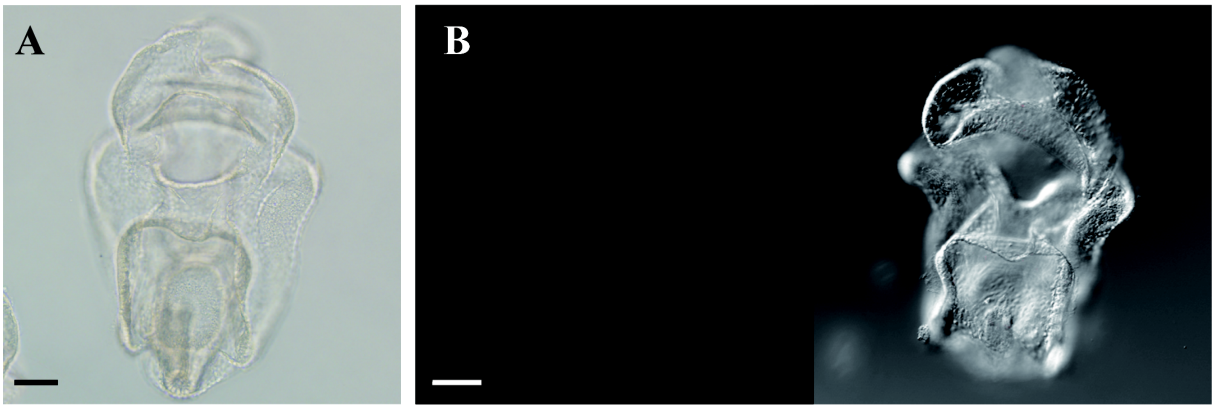

Supplementary Fig. S2. Whole mount in situ hybridization (control experiment for examination of *PpTRPA1* expression). (A-C) A sense probe for *PpTRPA1* was used instead of an antisense one. 4-days-old bipinnaria larvae were used and the arrangement of the panels is the same as those in Fig. 4. No signal was seen. Scale bar, 50 µm.

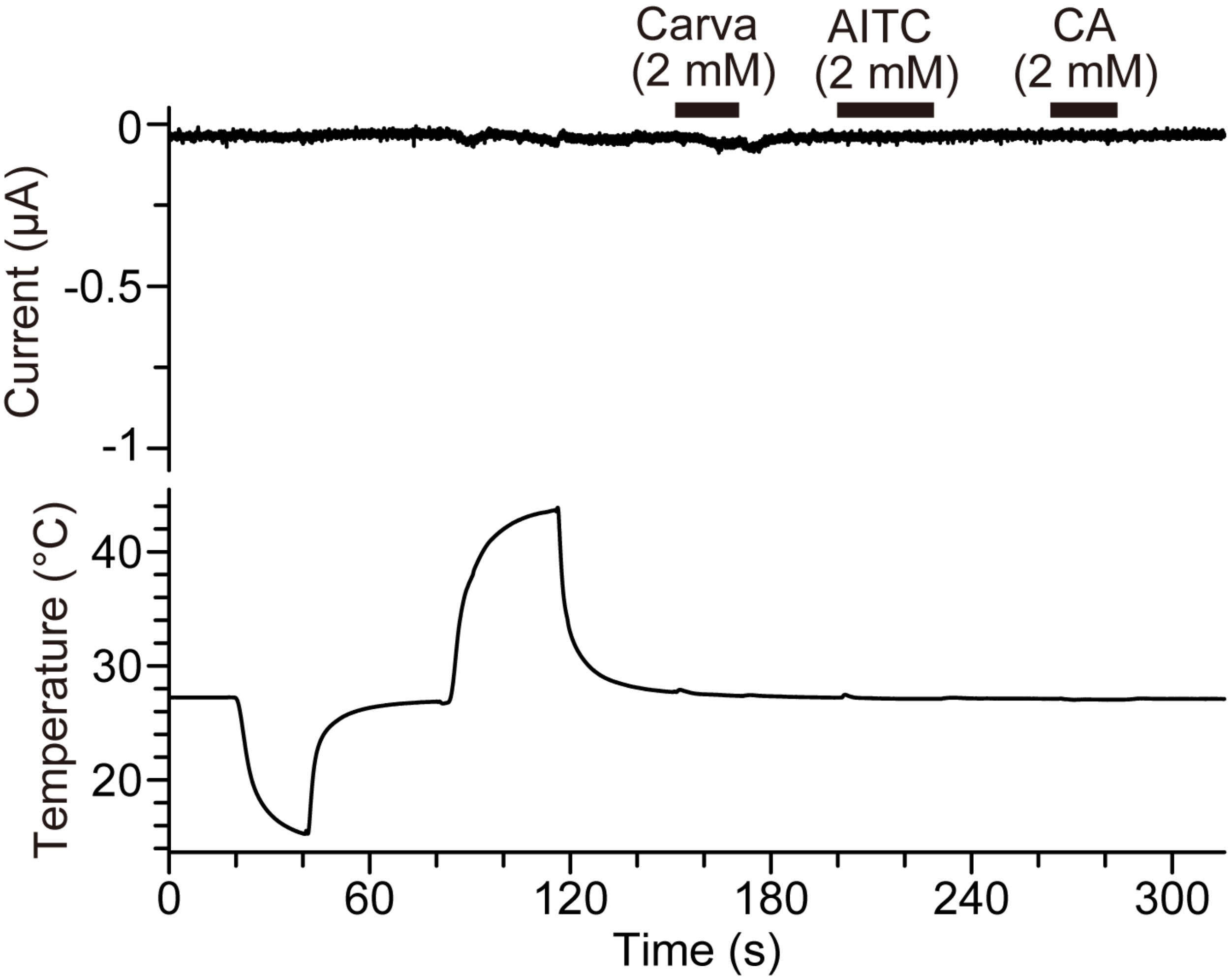

Supplementary Fig S3. Electrophysiological properties of *Pp*TRPA basal.

Representative current (upper) and temperature (lower) traces against cold, heat, carvacrol (Carva), AITC, and CA applications in *X. laevis* oocytes expressing *Pp*TRPA basal (n =3).

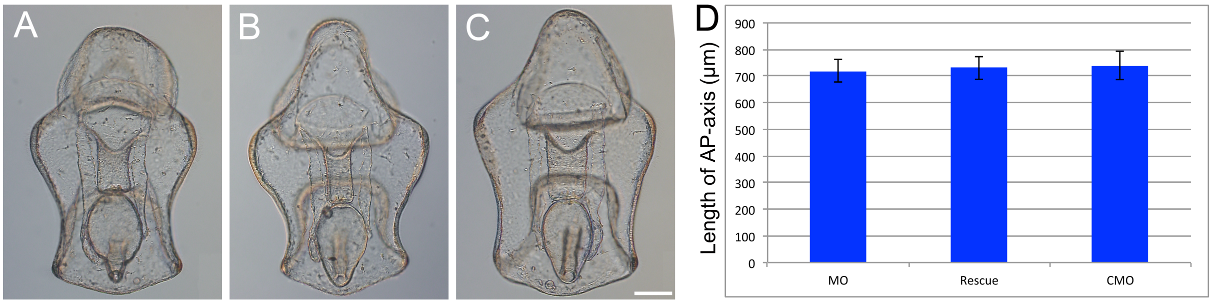

Supplementary Fig. S4. Morphology of (A) a *PpTRPA1* MO, (B) a rescued and (C) a CMO larva. Photographs were taken 4 days after fertilization. Scale bar, 100 µm. (**D**) Comparison of larval sizes among **(A)**, **(B)** and **(C)**. Graph shows the mean with s. d. of larval length along the anterior-posterior axis. Ten to twenty larvae were tested in three independent batches.

Supplementary movie legends

Supplementary movie 1. Thermotaxis assay in a 20 °C (right) to 25 °C (left) temperature gradient with normal bipinnaria larvae.

Supplementary movie 2. Thermotaxis assay without a thermal gradient with normal bipinnaria larvae.

Supplementary movie 3. Thermotaxis assay in a 26 °C (right) to 36 °C (left) temperature gradient with normal bipinnaria larvae.

Supplementary movie 4. Thermotaxis assay in a 20 °C (right) to 25 °C (left) temperature gradient with RR-treated bipinnaria larvae.

Supplementary movie 5. Thermotaxis assay in a 20 °C (right) to 25 °C (left) temperature gradient with a *Pp*TRPA1-morphant.

Supplementary movie 6. Thermotaxis assay in a 20 °C (right) to 25 °C (left) temperature gradient with rescued larvae.
